# Trends in hospital discharges, management and in-hospital mortality from acute myocardial infarction in Switzerland between 1998 and 2008

**DOI:** 10.1186/1471-2458-13-270

**Published:** 2013-03-25

**Authors:** Charlène Insam, Fred Paccaud, Pedro Marques-Vidal

**Affiliations:** 1 Institute of Social and Preventive Medicine (IUMSP), Lausanne University Hospital, Lausanne, Switzerland; 2 Institut Universitaire de Médecine Sociale et Préventive, Route de la Corniche 10, Lausanne, 1010, Switzerland

**Keywords:** Acute myocardial infarction, Coronary revascularization, Epidemiology, Switzerland, Trends, Drug-eluting stent, Coronary artery bypass graft, Hospital discharge

## Abstract

**Background:**

Since the late nineties, no study has assessed the trends in management and in-hospital outcome of acute myocardial infarction (AMI) in Switzerland. Our objective was to fill this gap.

**Methods:**

Swiss hospital discharge database for years 1998 to 2008. AMI was defined as a primary discharge diagnosis code I21 according to the ICD10 classification. Invasive treatments and overall in-hospital mortality were assessed.

**Results:**

Overall, 102,729 hospital discharges with a diagnosis of AMI were analyzed. The percentage of hospitalizations with a stay in an Intensive Care Unit decreased from 38.0% in 1998 to 36.2% in 2008 (p for trend < 0.001). Percutaneous revascularizations increased from 6.0% to 39.9% (p for trend < 0.001). Bare stents rose from 1.3% to 16.6% (p for trend < 0.001). Drug eluting stents appeared in 2004 and increased to 23.5% in 2008 (p for trend < 0.001). Coronary artery bypass graft increased from 1.0% to 3.0% (p for trend < 0.001). Circulatory assistance increased from 0.2% to 1.7% (p for trend < 0.001). Among patients managed in a single hospital (not transferred), seven-day and total in-hospital mortality decreased from 8.0% to 7.0% (p for trend < 0.01) and from 11.2% to 10.1%, respectively. These changes were no longer significant after multivariate adjustment for age, gender, region, revascularization procedures and transfer type. After multivariate adjustment, differing trends in revascularization procedures and in in-hospital mortality were found according to the geographical region considered.

**Conclusion:**

In Switzerland, a steep rise in hospital discharges and in revascularization procedures for AMI occurred between 1998 and 2008. The increase in revascularization procedures could explain the decrease in in-hospital mortality rates.

## Background

Cardiovascular Diseases (CVD) remain one of the major causes of deaths worldwide. Switzerland has one of the lowest CVD mortality rates within Europe [1] with a sustained downward trend over the last decades [2]. Acute myocardial infarction (AMI) can be treated with various drug therapies and revascularization interventions. Indeed, management of AMI, namely regarding revascularization interventions, has evolved over the last decades [3-8], following the continuously renewed guidelines [9,10].

There are few data on the evolution of AMI management and outcome in Switzerland. A study published in 2006 [8] based on data from 68 medical centers participating in the AMIS Plus register (http://www.amis-plus.ch/Project.htm) assessed Swiss trends in invasive treatment and outcome for the period 1997 to 2005. Still, it is unknown if the findings from this study also apply to non participating centers, and whether these trends are equally applied in all Swiss regions. Indeed, in Switzerland, health policies rarely go down to clinical guidelines and, if they exist, they are decided at the hospital or canton level, with little or no intervention from the federal bodies. The purpose of this study was to assess the trends in AMI management and outcome for the whole country and for the main administrative regions, using data from the Swiss hospital discharge database for the period 1998–2008.

## Methods

### Databases and available data

Data from the hospital discharge database for years 1998 to 2008 was used. The database was provided by the Swiss federal office of statistics (http://www.bfs.admin.ch) and covers 99% of public and private hospitals within Switzerland [11]. Data providing is compulsory and the information collected includes gender, age, length of stay, discharge status (main and secondary diagnoses, vital status) and procedures. Four types of stays could be obtained: “passing through”, patients who were admitted from another hospital and transferred to another hospital; “inbound”, patients who were admitted from another hospital and managed on-site; “outbound”, patients who were managed on-site and transferred to another hospital and “in-house”, patients who were admitted and managed without being transferred to another hospital.

Overall length of stay was indicated in days and length of stay in an intensive care unit (ICU) in hours. When the length of stay in the ICU was zero, it was considered as no stay in ICU. Vital status at discharge was indicated as dead or alive. Overall and seven-day in-hospital all-cause mortalities were computed; for seven-day mortality, patients who died after seven days were considered as being alive.

Main and secondary diagnoses at discharge were coded using the International Classification of Diseases, 10^th^ revision (ICD10) of the World Health Organization. Acute myocardial infarction was defined as ICD10 code I21X, where X = any number. The ICD10 coding does not distinguish between STEMI and non-STEMI. Procedures were coded using the International Classification of Diseases, 9^th^ Revision, Clinical Modification (ICD9-CM). The following revascularization procedures (and corresponding IDC9-CM codes) were assessed: any percutaneous coronary intervention (360X); bare (non-drug-eluting) stent (3606); drug-eluting stent (DES: 3607); coronary artery bypass grafting (CABG: 361X, 362X and 363X); circulatory assistance (376X and 369X) and thrombolysis (3602, 3604 and 991); and 379X for other procedures. All these procedures were coded as binary (yes/no) variables.

Inclusion criteria were: 1) Acute myocardial infarction (ICD10 code I21X) as the main discharge diagnosis and 2) age ≥18 years. Exclusion criterion was cardiovascular rehabilitation (ICD9-CM code Z500). Subsequent myocardial infarction (ICD10 code I22), was also excluded, as it was not possible to trace back to the initial event (and corresponding management) due to the anonymization of the data.

Population statistics by age were also obtained from the Swiss federal office of statistics and used to calculate a discharge rate, expressed as the number of discharges per 100,000 adult (≥18 years) inhabitants.

### Statistical analysis

Statistical analysis was conducted using SAS software version 9.2 (SAS Inc., Cary, NC, USA). Results were expressed as average ± standard deviation and as number of subjects and (percentage). Two sets of analyses were performed: the main analysis for trends in revascularization procedures included all hospital discharges, a supplementary analysis was carried out to limit the impact of transfers on significance levels and included only patients who were treated in one hospital (in-hospital).

Trends in the annual use of revascularisation procedures were assessed by Cochran-Armitage test for trend and further confirmed by multivariate logistic regression adjusting for age, gender and type of transfer; when assessing trends for Switzerland, a further adjustment was performed on region. Trends for seven-day and overall mortality rates were assessed among inbound and in-house patients only, as among “passing through” and outbound patients mortality was zero. Multivariate models assessing trends for seven-day and overall hospital mortality rates were adjusted for age, gender, ICU stay (yes/no), hemodynamic assistance (yes/no) and revascularization procedures (yes/no code: bare stent, drug eluting stent and CABG); when assessing trends for Switzerland, a further adjustment was performed on region. Results were expressed as Odds ratio (OR) and (95% confidence interval). Statistical significance was assessed for p < 0.05.

## Results

### Characteristics of the patients

Overall, data from 102,729 hospital discharges from AMI were analyzed. The characteristics of the patients discharged according to year are summarized in Table 1. The percentage of women tended to decrease, while the mean age of patients at discharge increased by one year. Discharge rates more than duplicated, from 98.2/100,000 inhabitants in 1998 to 221.3 in 2008 (Table 1).

**Table 1:** Characteristics of patients discharged from hospital with a main diagnosis of acute myocardial infarction, Switzerland, for period 1998–2008

**Year**	**Number of discharges**	**Women (%)**	**Age (years)**	**Rate §**
**1998**	5,530	31.8	66.5 ± 13.9	98.2
**1999**	6,731	31.4	66.9 ± 13.7	118.8
**2000**	7,462	31.4	67.2 ± 13.6	130.7
**2001**	8,235	32.4	67.0 ± 14.0	142.5
**2002**	9,004	31.3	67.0 ± 14.0	154.2
**2003**	8,987	31.2	67.2 ± 14.2	152.5
**2004**	9,251	30.8	66.8 ± 14.1	155.5
**2005**	9,804	30.3	67.3 ± 14.0	163.5
**2006**	11,521	29.2	66.9 ± 13.9	190.3
**2007**	12,370	29.9	67.3 ± 13.9	201.4
**2008**	13,834	31.3	67.6 ± 13.8	221.3

Results are expressed as percentage or as mean ± standard deviation. Trends assessed by Cochran-Armitage test or linear regression: p < 0.001 for gender and age. § defined as the ratio between the number of discharges and the adult (≥18 years) population, expressed per 100,000 inhabitants.

**Figure 1:**
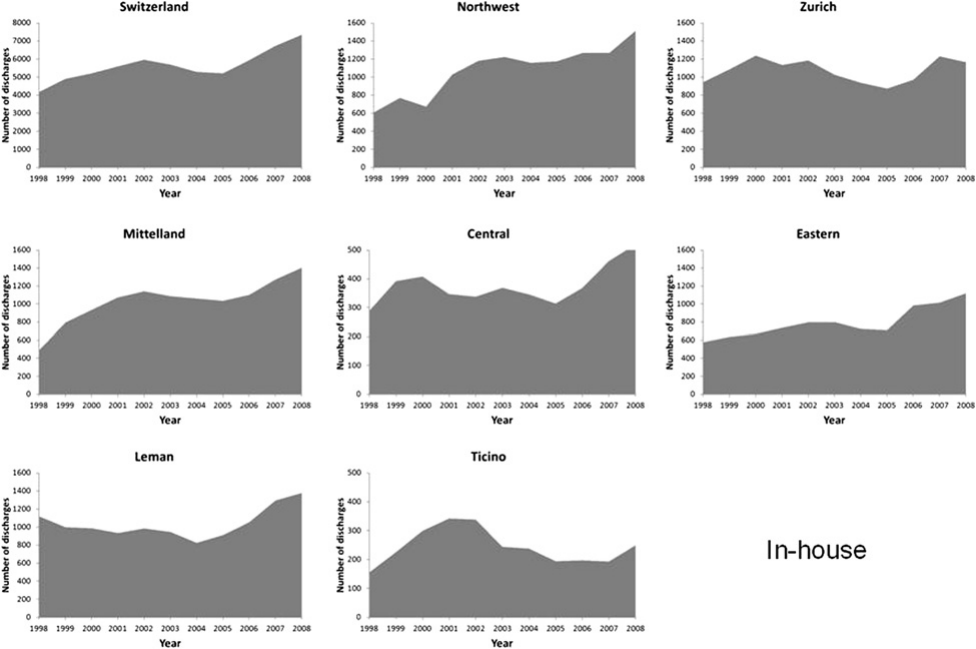
Number of patients discharged with a main diagnosis of acute myocardial infarction who were managed in a single hospital (in-house), in Switzerland and by region, 1998–2008.

**Figure 2:**
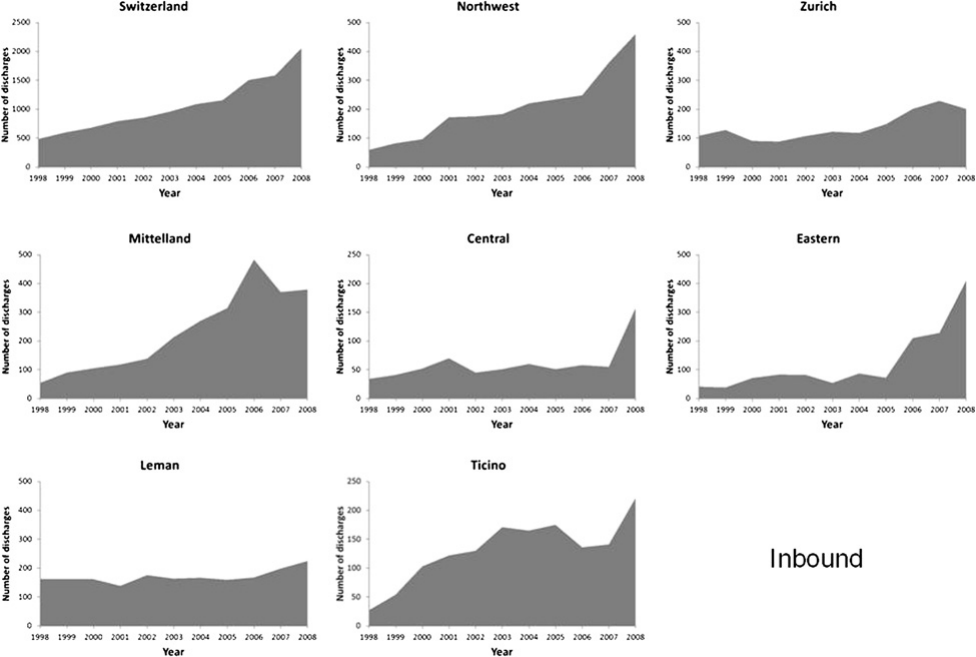
Number of patients with a main diagnosis of acute myocardial infarction who were admitted from another hospital and managed on-site (inbound), in Switzerland and by region, 1998–2008.

**Figure 3:**
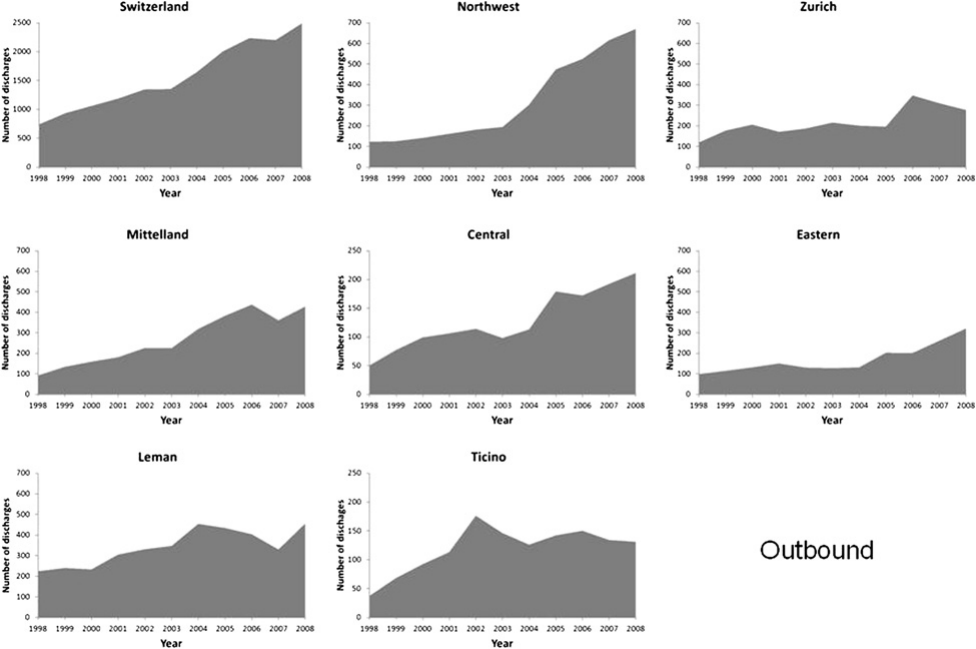
Number of patients with a main diagnosis of acute myocardial infarction who were managed on-site and transferred to another hospital (outbound), in Switzerland and by region, 1998–2008.

**Figure 4:**
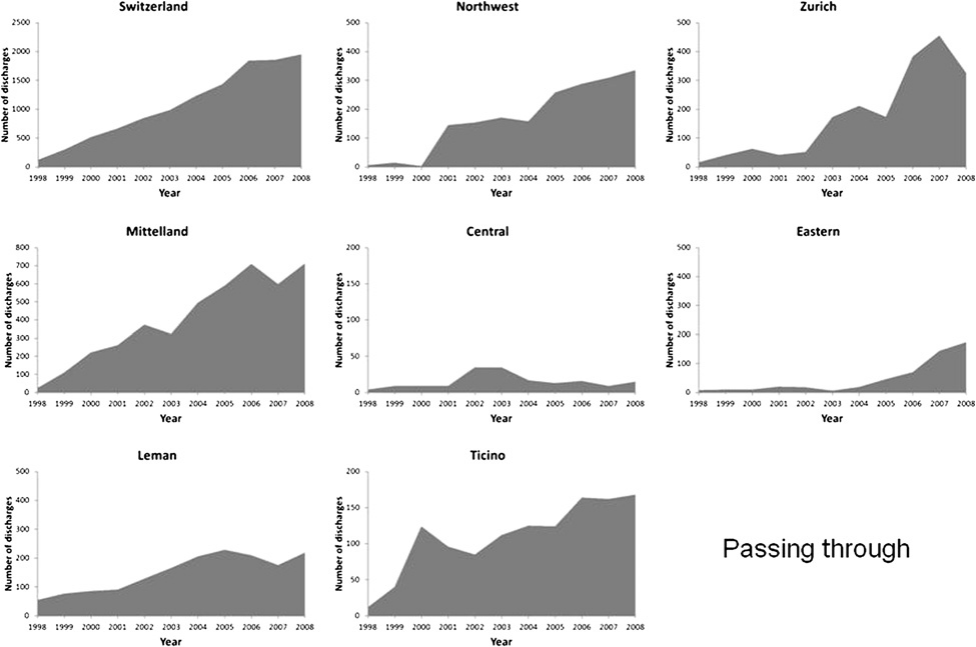
Number of patients with a main diagnosis of acute myocardial admitted from another hospital and transferred to another hospital (passing through), in Switzerland and by region, 1998–2008.

The number of discharges from AMI rose considerably from 5530 in 1998 to 13,834 in 2008 (Figures 1, 2, 3 and 4). Most of this increase was due to between-hospital transfers (inbound, outbound and passing through, Figures 2, 3 and 4), which increased from 24% in 1998 to 47% in 2008 (for Switzerland). In 2008, over half of hospital discharges for AMI were due to transfers in Mittelland (52%) and Ticino (68%).

### Intensive care unit

**Figure 5:**
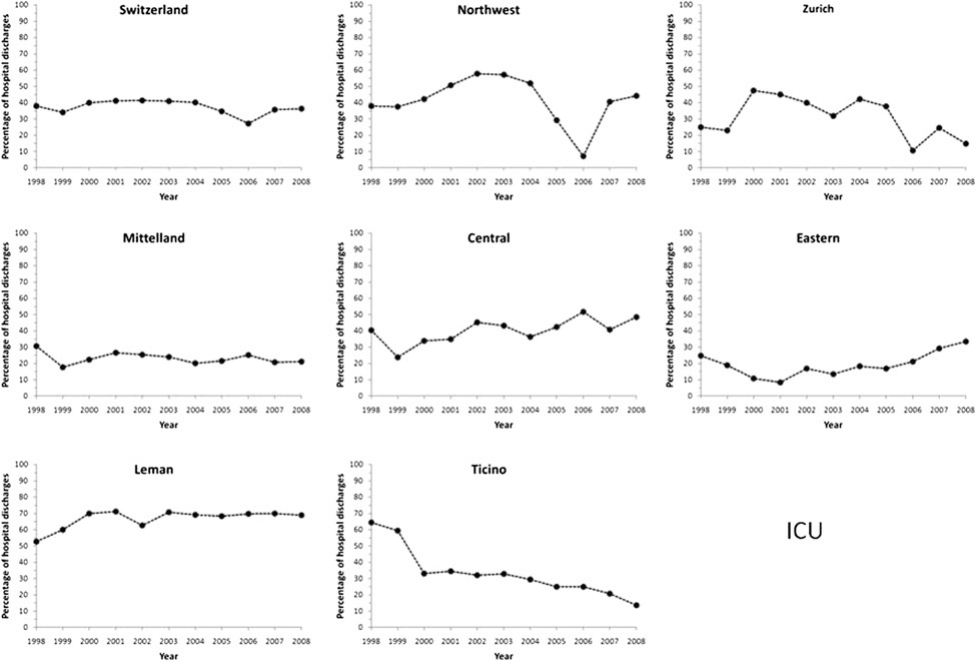
**Trends in intensive care unit (ICU) utilization for acute myocardial infarction in Switzerland, overall and by region, for the period 1998–2008.** Results are expressed as percentage of hospital discharges with a diagnosis of acute myocardial infarction.

The percentage of hospitalizations with a stay in an Intensive Care Unit decreased from 38.0% in 1998 to 36.2% in 2008 (p for trend < 0.001, Figure 5). This trend was further confirmed after multivariate adjustment for Switzerland. Differing trends were found according to the region considered: increase in Leman, Eastern and Central Switzerland; decrease in Northwest, Zurich and Ticino and no change in Mittelland (Table 2). Restricting the analysis to patients managed in a single hospital led to similar results (Additional file 1: Figure S1).

**Table 2:** Multivariate analysis of the trends in revascularisation procedures and in in-hospital mortality for patients discharged from hospital with a main diagnosis of acute myocardial infarction, Switzerland and Swiss regions, for period 1998–2008

	**Switzerland**	**Leman**	**Mittelland**	**Northwest**	**Zurich**	**Eastern**	**Central**	**Ticino**
Revascularization procedures								
Number of discharges, all	102,729	18,702	21,275	19,492	17,684	12,522	6,408	6,646
ICU	0.99 (0.98–1.00)	1.06 (1.05–1.07)	0.99 (0.98–1.01)	0.94 (0.93–0.95)	0.92 (0.91–0.93)	1.10 (1.08–1.11)	1.10 (1.08–1.12)	0.85 (0.83–0.87)
Angioplasty	1.25 (1.24–1.25)	1.20 (1.19–1.21)	1.27 (1.25–1.28)	1.26 (1.24–1.27)	1.21 (1.19–1.22)	1.60 (1.55–1.64)	1.80 (1.72–1.89)	1.10 (1.08–1.12)
Bare stent	1.11 (1.10–1.12)	1.14 (1.13–1.15)	1.08 (1.06–1.10)	1.06 (1.04–1.08)	1.07 (1.06–1.08)	1.31 (1.26–1.36)	1.62 (1.54–1.69)	1.05 (1.02–1.07)
Drug-eluting stent	1.66 (1.65–1.68)	1.59 (1.55–1.64)	1.54 (1.51–1.58)	1.52 (1.49–1.56)	1.96 (1.88–2.03)	2.14 (2.03–2.26)	2.97 (2.54–3.46)	2.58 (2.38–2.80)
CABG	1.08 (1.06–1.09)	1.06 (1.03–1.09)	1.19 (1.15–1.23)	1.07 (1.03–1.10)	1.03 (1.00–1.06)	1.71 (1.25–2.33)	DNC	1.02 (0.98–1.06)
Circulatory assistance	1.17 (1.14–1.19)	1.10 (1.06–1.14)	1.27 (1.20–1.35)	1.24 (1.15–1.33)	1.13 (1.10–1.17)	2.09 (1.59–2.74)	1.92 (1.45–2.54)	1.02 (0.88–1.19)
Thrombolysis	1.00 (0.99–1.02)	1.02 (1.00–1.05)	0.99 (0.97–1.02)	0.92 (0.88–0.95)	1.06 (1.03–1.08)	1.09 (1.04–1.14)	0.96 (0.90–1.02)	0.91 (0.87–0.95)
In-hospital mortality								
Number of discharges §	73,764	13,301	13,905	14,143	13,338	10,132	4,826	4,119
Seven-day mortality	1.00 (0.99–1.01)	1.00 (0.98–1.02)	1.02 (1.00–1.04)	0.99 (0.97–1.01)	0.99 (0.97–1.01)	0.99 (0.96–1.01)	1.03 (0.99–1.06)	1.02 (0.98–1.06)
Overall mortality	1.00 (0.99–1.01)	1.00 (0.98–1.02)	1.02 (1.00–1.04)	1.00 (0.98–1.02)	0.99 (0.97–1.01)	0.98 (0.96–0.99)	1.03 (1.00–1.06)	1.02 (0.99–1.06)
Number of discharges §§	62,021	11,417	11,371	11,853	11,798	8756	4152	2674
Seven-day mortality	1.01 (1.00–1.02)	1.00 (0.98–1.03)	1.03 (1.01–1.05)	1.00 (0.97–1.02)	1.00 (0.98–1.02)	0.99 (0.97–1.02)	1.05 (1.01–1.09)	DNC
Overall mortality	1.01 (1.00–1.02)	1.01 (0.99–1.03)	1.03 (1.01–1.05)	1.01 (0.99–1.03)	1.00 (0.98–1.01)	0.99 (0.96–1.01)	1.04 (1.01–1.08)	1.03 (0.99–1.07)

Results are expressed as multivariate adjusted Odds ratio and (95% confidence interval) for a one-year increase. *ICU* intensive care unit; *CABG* coronary artery bypass graft; *DNC* model did not converge. Statistical analysis by multivariate logistic regression. For revascularization procedures, analysis was conducted adjusting for age (continuous), gender and transfer type (in-house, inbound, outbound and passing through) for each Swiss region; for Switzerland, a further adjustment was performed on region. For in-hospital mortality, analysis was conducted adjusting for age (continuous), gender, ICU stay (yes/no), hemodynamic assistance (yes/no) and revascularization procedures (yes/no code: bare stent, drug eluting stent and CABG) for each Swiss region; for Switzerland, a further adjustment was performed on region. §, patients not transferred to another hospital (inboud or in-house); §§, patients managed in a single hospital (in-house).

### Revascularisation procedures

**Figure 6:**
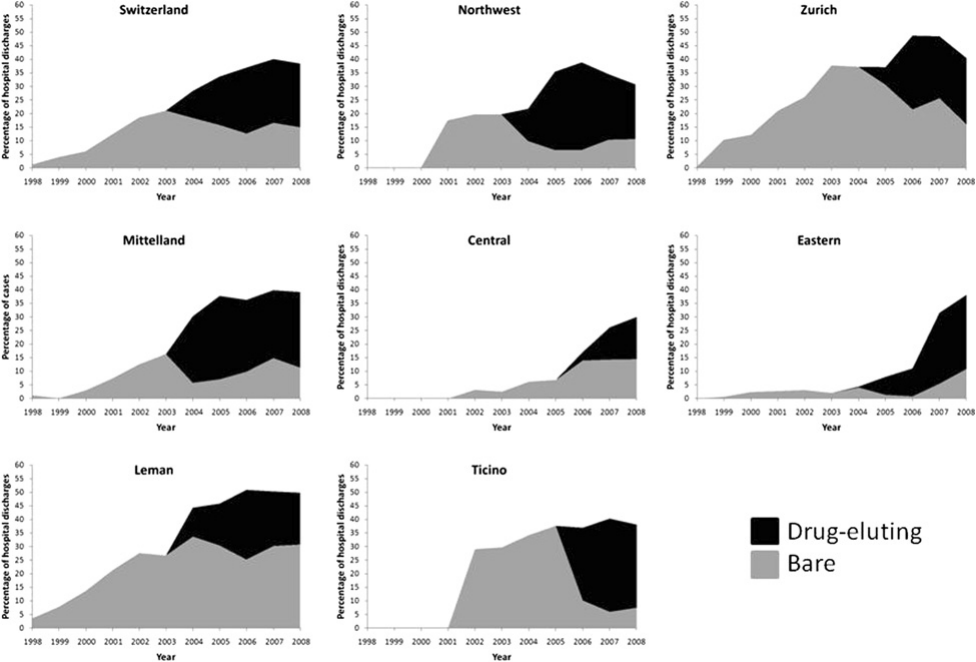
**Trends in the use of drug-eluting and non-drug-eluting stents for acute myocardial infarction in Switzerland, overall and by region, for the period 1998–2008.** Results are expressed as percentage of hospital discharges with a diagnosis of acute myocardial infarction.

Percutaneous revascularizations increased sevenfold, from 6.0% of all patients discharged with AMI in 1998 to 42.4% in 2006, with a slight decrease to 39.9% in 2008 (p for trend < 0.001, Figure 6). This trend was further confirmed after multivariate adjustment for Switzerland and all regions considered (Table 2).

Bare (non-drug-eluting) stents rose from 1.3% in 1998 to peak at 21.1% in 2003, further decreasing to 16.6% in 2008 (p for trend < 0.001, Figure 6). This trend was further confirmed after multivariate adjustment for Switzerland and all Swiss regions considered (Table 2).

DES appeared in 2004 and steadily peaked at 24.3% in 2006, with a slight decrease to 23.5% in 2008 (p for trend < 0.001, Figure 6). This trend was further confirmed after multivariate adjustment for Switzerland and all regions concerned, although stronger for Central, Ticino, Eastern and Zurich than for Leman, Mittelland or Northwest (Table 2). Restricting the analysis to patients managed in a single hospital led to similar results (Additional file 2: Figure S2).

**Figure 7:**
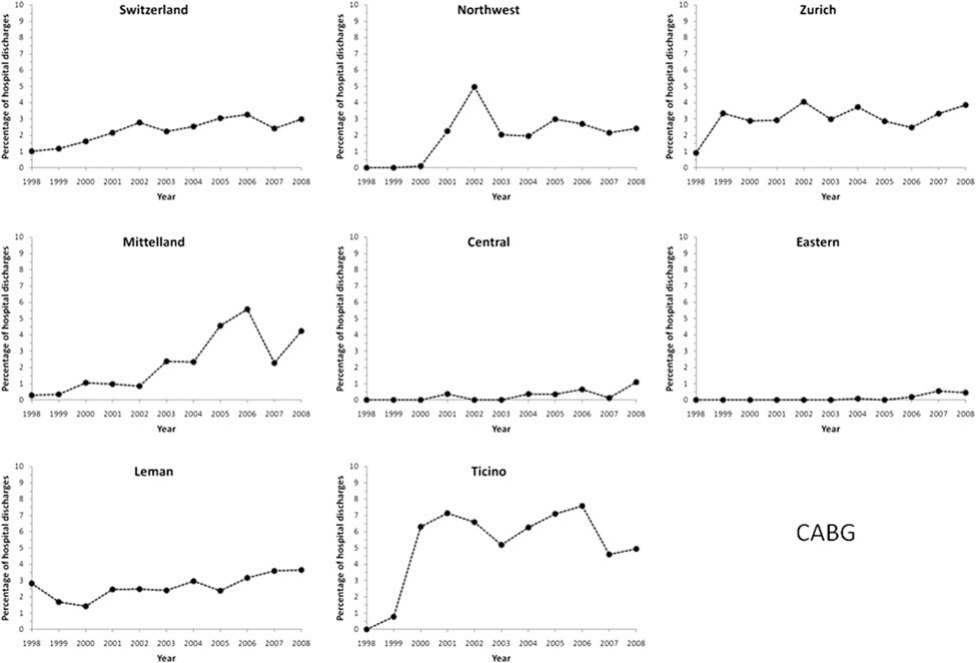
**Trends in the use of coronary artery bypass graft (CABG) for acute myocardial infarction in Switzerland, overall and by region, for the period 1998–2008.** Results are expressed as percentage of hospital discharges with a diagnosis of acute myocardial infarction.

CABG increased from 1.0% in 1998 to peak at 3.3% in 2006, further decreasing to 3.0% in 2008 (p for trend < 0.001, Figure 7). This trend was further confirmed after multivariate adjustment for Switzerland and all Swiss regions, with the exception of Ticino (Table 2). Restricting the analysis to patients managed in a single hospital led to similar results (Additional file 3: Figure S3).

Circulatory assistance increased from 0.24% in 1998 to 2.00% in 2007 and decreased to 1.71% in 2008 (p for trend < 0.001). This trend was further confirmed after multivariate adjustment for Switzerland and all Swiss regions, with the exception of Ticino (Table 2).

Finally, no significant trends were found for thrombolysis, which increased from 0.5% in 1998 to 6.0% in 2002 and decreased to 1.9% in 2008 (p for trend = 0.09). This trend was further confirmed after multivariate adjustment for Switzerland. Conversely, differing trends were found according to the region considered: increase in Leman, Zurich and Eastern Switzerland, decrease in Northwest and Ticino and no change in Mittelland and Central Switzerland (Table 2).

### In-hospital mortality

**Figure 8:**
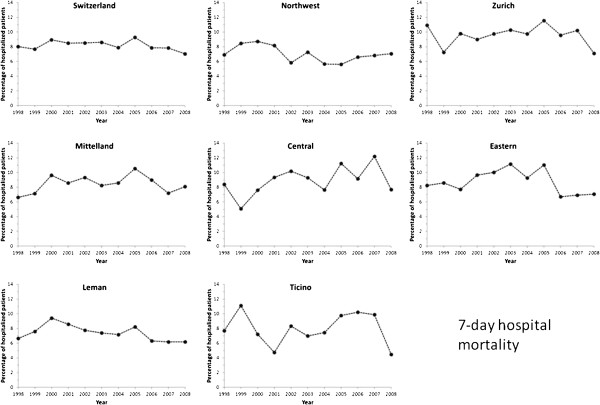
**Trends in seven-day in-hospital mortality rates from acute myocardial infarction in Switzerland, overall and by region, for the period 1998–2008.** Results are expressed as percentage of patients inbound or in-house discharged with a diagnosis of acute myocardial infarction.

**Figure 9:**
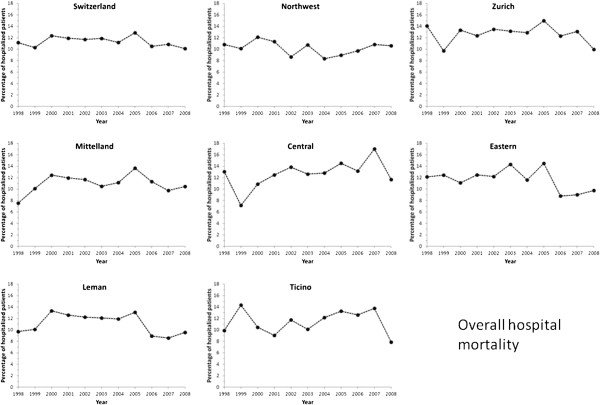
**Trends in overall in-hospital mortality rates from acute myocardial infarction in Switzerland, overall and by region, for the period 1998–2008.** Results are expressed as percentage of patients inbound or in-house discharged with a diagnosis of acute myocardial infarction.

Among patients not transferred to another hospital, seven-day in-hospital mortality decreased from 8.0% in 1998 to 7.0% in 2008 (p for trend < 0.01, Figure 8), while total hospital mortality decreased from 11.2% in 1998 to 10.1% in 2008 (p for trend < 0.01, Figure 9). After multivariate adjustment on age, gender, ICU and revascularization procedures, no change in seven-day in-hospital mortality was found for Switzerland and all Swiss regions (Table 2). Restricting the analysis to patients managed in a single hospital led to similar results, with the exception of an increase in mortality in Mittelland and Central Switzerland (Table 2 and Additional file 4: Figure S4).

For overall in-hospital death, no significant trend was found for Switzerland, while differing trends were found for regions: decrease in Eastern; increase in Mittelland and Central and no change in Leman, Northwest, Zurich and Ticino (Table 2). Restricting the analysis to patients managed in a single hospital led to similar results, except that the decrease in Eastern Switzerland was no longer statistically significant (Table 2).

## Discussion

Since the end of the MONICA study [12,13], few studies have assessed the management and outcome of AMI in Switzerland. Our results show an increase in most revascularization procedures, namely PCI, while CABG tended to stabilize or even to decrease in the most recent years. Moreover, these trends strongly differ according to region. Overall, the changes in revascularization procedure in Switzerland follow the ones observed in other countries, [6,7,14,15]. These findings are also in agreement with a previous study conducted on 19,500 patients of the AMIS Plus registry [8], although the increase in PCI procedures observed [16] was considerably stronger than in ours. This difference is most likely due to the overrepresentation of PCI procedures among the AMIS Plus registry hospitals as the Registry includes only selected voluntary teams.

Between 1998 and 2008, the number of hospital discharges for AMI increased over two-fold in Switzerland. This finding is in contradiction with some previous studies [17,18], but it should be noted that these studies showed declining rates of AMI rather than number of discharges. Part of this increase might be due to population aging [2], although the observed increase in population age (from 66.5 years in 1998 to 67.6 years in 2008) would not lead to a doubling of the number of hospitalizations for AMI. Further, all analyses were adjusted for age. Another possibility would be a change in disease coding, but again this is unlikely as hospital discharge data has been shown to be reliable regarding the diagnosis of AMI [19] and as this study focused on a specific code. It is also possible that more people are hospitalized for a second or even a third AMI (partly related to the decrease in case fatality rate of the first AMI, leading to an increased incidence of 2^nd^ or 3^rd^ AMI). Another possibility is the change in criteria defining AMI [20], which has been shown to increase the incidence of AMI [21]. The most likely explanation for the considerable increase in the number of hospital discharges for AMI in Switzerland is the steep rise in hospital transfers, which accounted for almost half of hospital discharges for AMI in 2008. The increase in hospital transfers might be due to the limited number of hospitals capable of performing revascularization procedures, prompting smaller hospitals to transfer their patients as recommended by European guidelines [22,23]. Still, considerable differences in the percentages of hospital discharges due to transfers were noted: in 2008 the values ranged from 40% in Leman to 68% in Ticino. These differences might be due to local policies. As AMI management is expensive [24], the increase in hospital discharges for AMI would add a considerable pressure in Swiss health expenditures, which already increased from 9.6% in 1995 to 11.4% of the Swiss GNP in 2009 [25].

### Intensive care unit

The percentage of hospitalizations with ICU stay remained stable between 1998 and 2008 in Switzerland. This stabilization might be the consequence of improved management, requiring less ICU admissions before or after a revascularization intervention. Another likely explanation is the increasing number of transfers, the patients being stabilized before being transferred to another hospital for revascularization. Still, considerable differences in the proportion of hospitalizations with ICU and corresponding trends were found between regions, suggesting that other factors such as the number of ICU beds available or local options for AMI management might be at play. Unfortunately there is no available information regarding the number of ICU beds in each region, so the precise reasons for the regional differences in ICU admissions for AMI remain to be further assessed.

### Revascularisation procedures

Stent use increased steeply from slightly over 1% in 1998 to nearly 40% in 2008, a finding in agreement with the literature [26]. DES appeared in 2004 and rose steadily until 2006, when several reports questioning their costs [27,28] and long term complications [29,30] were published. These reports slowed down DES use, but some Swiss regions were more responsive than others. For instance, in the Leman area DES use equaled but never exceeded bare stent use, while in South Switzerland (Ticino) DES represented nearly all stents implanted since 2005. The reasons for such regional differences are more likely due to local preferences or to local agreements regarding DES cost than to decisions based on scientific evidence.

The rate of CABG remained stable at 3% throughout the study period, a finding also reported in other countries [3,6]. As for stents, very different trends according to the region were found. For instance, South Switzerland (Ticino) showed a steep rise in CABG use, starting in 1999 to reach an average rate of 7%, well above the Swiss average. This trend might partly be explained by the creation in 1999 of new medical centers with the capacity to carry out these interventions. Conversely, in Eastern and Central Switzerland, hardly any CABG was performed until 2007, likely the result of the lack of required infrastructure. Overall, our results show that CABG rates evolved differently between Swiss regions, the most likely explanations being the existence of infrastructures and local preferences for certain types of revascularization procedures.

### In-hospital mortality

An overall decrease in seven-day in-hospital mortality was found for Switzerland, a finding also reported elsewhere [8,16]. This trend was no longer significant after multivariate adjustment, suggesting that most of the decrease in mortality is due to revascularization interventions and the improved quality of treatment according to the newest guidelines [9,10]. Restricting the analysis to patients who survived at least three days led to similar findings, although some regions presented a slight increase in mortality (Additional file 5: Table S1). The rising prevalence of CV risk factors in Switzerland [31] could also contribute to lessen the decrease in AMI mortality [32], but it was not possible to adequately consider them in the multivariate model as they are not reported systematically in the database. Similarly, it was not possible to assess whether the patients presented with a STEMI or a non-STEMI, and which drug treatments were provided during hospital stay, all factors associated with in-hospital mortality [22,23,33]. Overall, our data suggest that the increasing number of revascularization interventions performed among patients discharged with a main diagnosis of AMI do not suffice to decrease in-hospital mortality in Switzerland, a country presenting the lowest CVD mortality within Europe [1]. Further studies are mandatory to better assess the factors associated with in-hospital mortality from AMI in order to optimize patient management.

### Study limitations

This study has some limitations. Medicines are not included in the hospital discharge database, thus precluding the assessment of their trends. This absence might also explain the very low rates for thrombolysis, which are likely due to the absence of coding rather than a true absence of use. Furthermore, we lack data to assess whether the changes in thrombolytic therapy are related to changes in reporting levels or are actually real changes. The ICD10 coding does not distinguish STEMI from NSTEMI; hence, it is possible that part of the increase in discharges from AMI during the study period might be related to the change in the definition of NSTEMI populations due to the generalization of the use of troponin measurements. Indeed, introduction of troponin measurements led to the reclassification of many unstable angina patients into NSTEMI patients, with a 33% increase of the population of NSTEMIs from before to after the introduction of troponin measurements [34]. Still, including unstable angina (ICD10 code I200) in the analysis led to similar findings, with the exception that the increase in CABG was no longer significant and even decreased in some regions (Additional file 5: Table S1). The strong increase in the number of between-hospital transfers and the anonymization of the data (which precluded the follow-up of the patients) complicated the interpretation of the trends. Still, restricting the analysis to patients fully managed in a single hospital led to similar results. It was not possible to assess “true” seven- and 30-day mortality rates as no follow-up data was available for the patients discharged. The AMIS registry, which collects vital status data for AMI patients hospitalized in the participating institutions, will be able to provide such information [35]. Finally, there is no published information on the validity of hospital discharge data in Switzerland regarding the diagnosis of AMI. Still, using the results from an ongoing study (CoLaus), of 159 hospitalizations for AMI, 145 (91%) were confirmed as such by an independent panel of cardiologists, a value in agreement with the literature [36,37].

## Conclusion

In Switzerland, a steep rise in hospital discharges and in revascularization procedures for AMI occurred between 1998 and 2008.

## Competing interests

The authors declare that they have no competing interests.

## Authors’ contributions

CI made part of the statistical analyses and wrote most of the article; PMV collected data, made part of the statistical analysis and wrote part of the article; FP revised the article for important intellectual content. PMV had full access to the data and is the guarantor of the study. All authors read and approved the final manuscript.

## Pre-publication history

The pre-publication history for this paper can be accessed here:

http://www.biomedcentral.com/1471-2458/13/270/prepub

## Supplementary Material

Additional file 1: Figure S1 Trends in intensive care unit (ICU) utilization for acute myocardial infarction in Switzerland, overall and by region, for the period 1998–2008, patients managed in a single hospital (in-house). Results are expressed as percentage of patients discharged with a diagnosis of acute myocardial infarction. Click here for file

Additional file 2: Figure S2 Trends in the use of drug-eluting and non-drug-eluting stents for acute myocardial infarction in Switzerland, overall and by region, for the period 1998–2008, patients managed in a single hospital (in-house). Results are expressed as percentage of patients discharged with a diagnosis of acute myocardial infarction. Click here for file

Additional file 3: Figure S3 Trends in the use of coronary artery bypass graft (CABG) for acute myocardial infarction in Switzerland, overall and by region, for the period 1998–2008, patients managed in a single hospital (in-house). Results are expressed as percentage of patients discharged with a diagnosis of acute myocardial infarction. Click here for file

Additional file 4: Table S4 Trends in seven-day in-hospital mortality rates from acute myocardial infarction in Switzerland, overall and by region, for the period 1998–2008, patients managed in a single hospital (in-house). Results are expressed as percentage of patients discharged with a diagnosis of acute myocardial infarction. Click here for file

Additional file 5: Table S1. Trends in seven-day and overall in-hospital mortality, Switzerland and Swiss regions, for period 1998-2008 Click here for file
