# Supplementary figures and images for: Trends in hospital discharges, management and in-hospital mortality from acute myocardial infarction in Switzerland between 1998 and 2008
Source: BMC Public Health. 2013 Mar 25;13:270. doi: 10.1186/1471-2458-13-270 (PMC3626665; doi:10.1186/1471-2458-13-270)

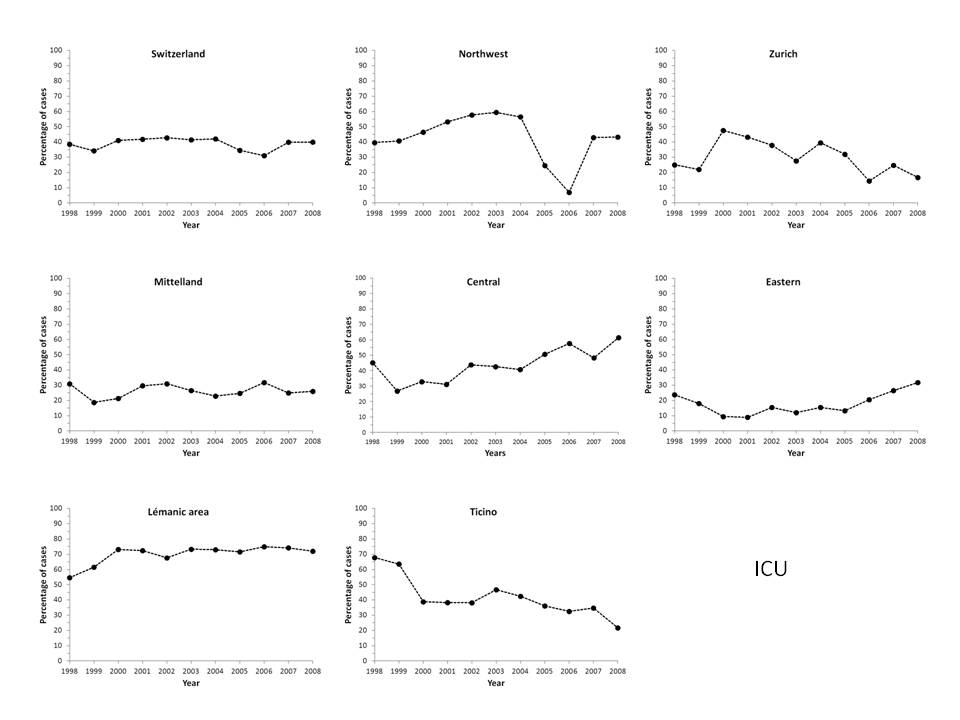

Supplement: Additional file 1: Figure S1 — Trends in intensive care unit (ICU) utilization for acute myocardial infarction in Switzerland, overall and by region, for the period 1998–2008, patients managed in a single hospital (in-house). Results are expressed as percentage of patients discharged with a diagnosis of acute myocardial infarction. [file 1471-2458-13-270-S1.jpeg]

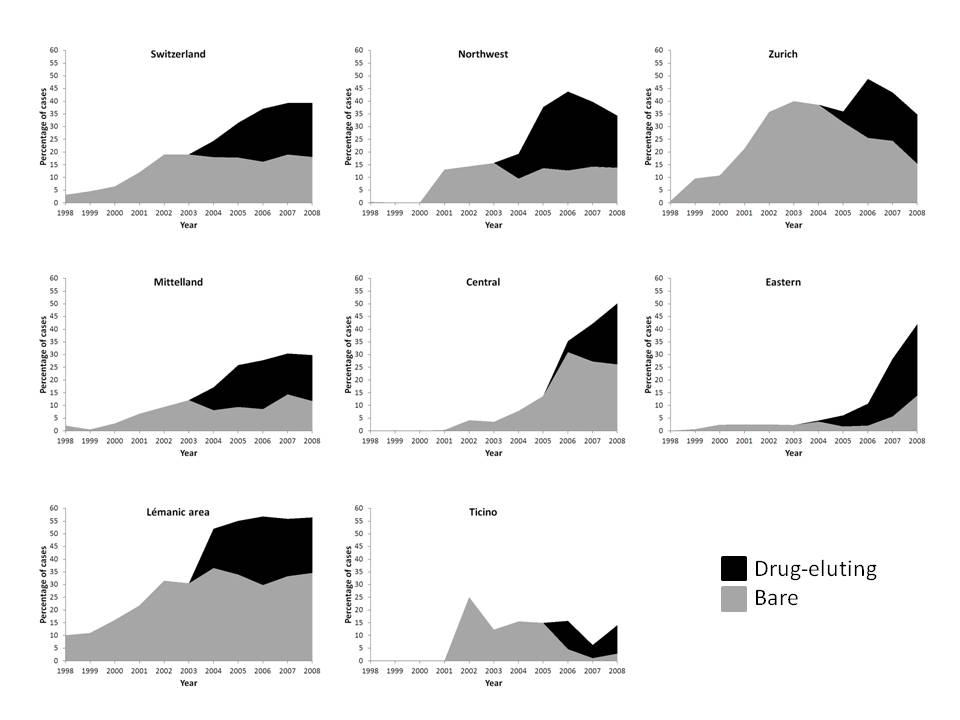

Supplement: Additional file 2: Figure S2 — Trends in the use of drug-eluting and non-drug-eluting stents for acute myocardial infarction in Switzerland, overall and by region, for the period 1998–2008, patients managed in a single hospital (in-house). Results are expressed as percentage of patients discharged with a diagnosis of acute myocardial infarction. [file 1471-2458-13-270-S2.jpeg]

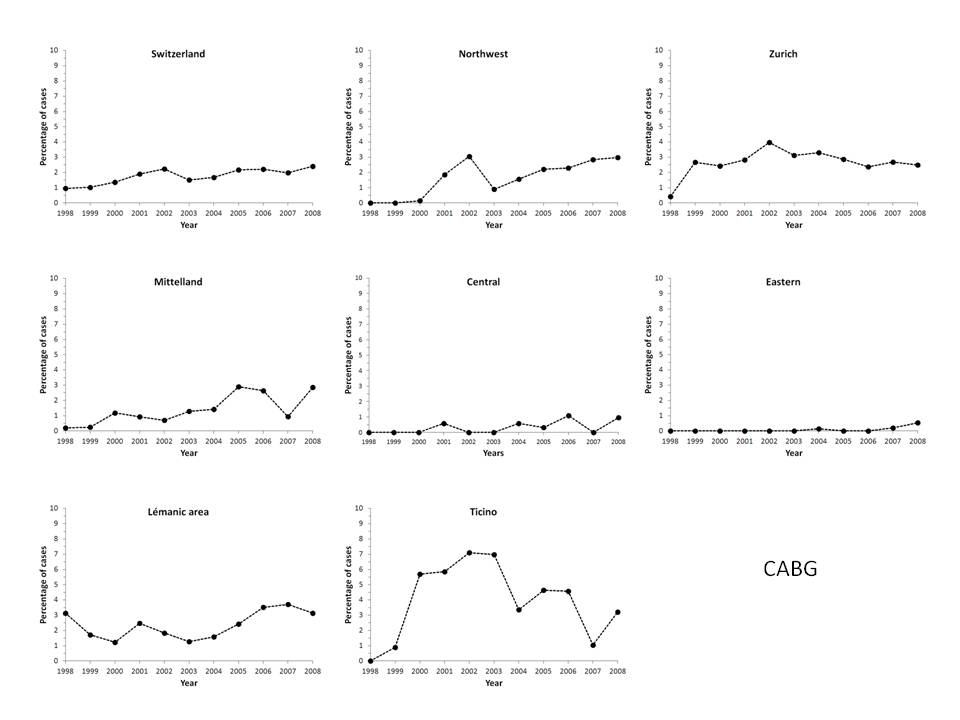

Supplement: Additional file 3: Figure S3 — Trends in the use of coronary artery bypass graft (CABG) for acute myocardial infarction in Switzerland, overall and by region, for the period 1998–2008, patients managed in a single hospital (in-house). Results are expressed as percentage of patients discharged with a diagnosis of acute myocardial infarction. [file 1471-2458-13-270-S3.jpeg]

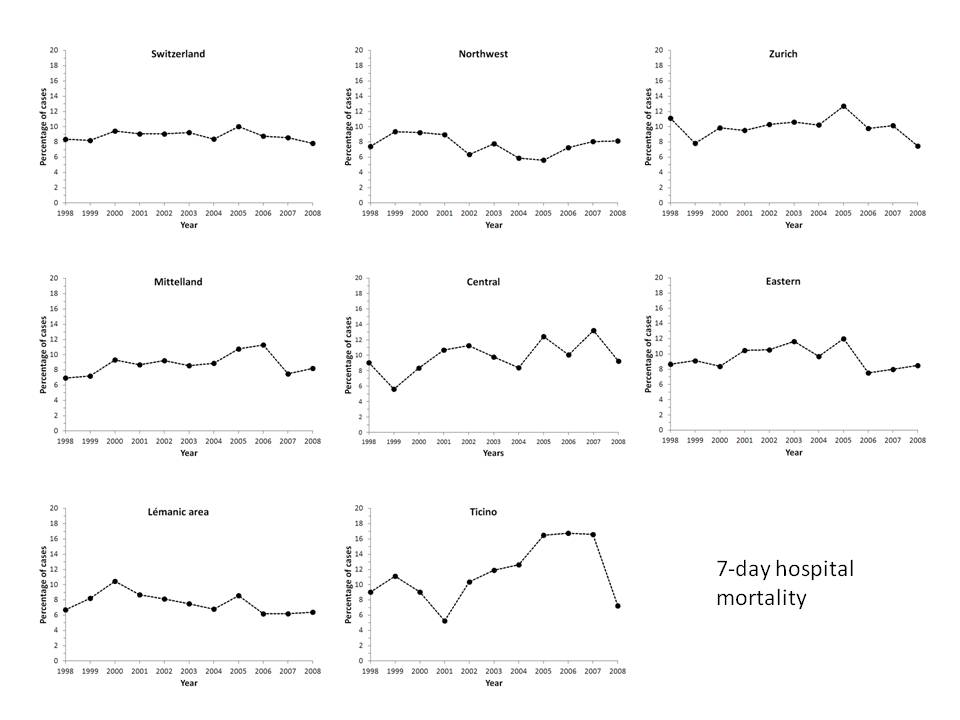

Supplement: Additional file 5: Table S1. — Trends in seven-day and overall in-hospital mortality, Switzerland and Swiss regions, for period 1998-2008 [file 1471-2458-13-270-S5.jpeg]
