# Supplementary material for: Trends in hospital discharges, management and in-hospital mortality from acute myocardial infarction in Switzerland between 1998 and 2008
Source: BMC Public Health. 2013 Mar 25;13:270. doi: 10.1186/1471-2458-13-270 (PMC3626665; doi:10.1186/1471-2458-13-270)
Supplement: Additional file 4: Table S4 — Trends in seven-day in-hospital mortality rates from acute myocardial infarction in Switzerland, overall and by region, for the period 1998–2008, patients managed in a single hospital (in-house). Results are expressed as percentage of patients discharged with a diagnosis of acute myocardial infarction. [file 1471-2458-13-270-S4.doc]

**Supplementary table 1**: trends in seven-day and overall in-hospital mortality, Switzerland and Swiss regions, for period 1998-2008.

|  | **Switzerland** | **Leman** | **Mittelland** | **Northwest** | **Zurich** | **Eastern** | **Central** | **Ticino** |
| --- | --- | --- | --- | --- | --- | --- | --- | --- |
| Number of patients | 62,021 | 11,417 | 11,371 | 11,853 | 11,798 | 8756 | 4152 | 2674 |
| Seven-day | 1.01 (1.00-1.02) | 1.00 (0.98-1.03) | 1.03 (1.01-1.05) | 1.00 (0.97-1.02) | 1.00 (0.98-1.02) | 0.99 (0.97-1.02) | 1.05 (1.01-1.09) | DNC |
| Overall | 1.01 (1.00-1.02) | 1.01 (0.99-1.03) | 1.03 (1.01-1.05) | 1.01 (0.99-1.03) | 1.00 (0.98-1.01) | 0.99 (0.96-1.01) | 1.04 (1.01-1.08) | 1.03 (0.99-1.07) |

Analysis restricted to patients discharged with a diagnosis of acute myocardial infarction managed in a single hospital. Results are expressed as Odds ratio and (95% confidence interval). Statistical analysis by multivariate logistic regression adjusting for age (continuous), gender, intensive care unit (yes/no, hemodynamic assistance (yes/no) and revascularisation procedures (yes/no: bare stent, drug eluting stent and CABG); when assessing trends for Switzerland, a further adjustment was performed on region. DNC: model did not converge.

**Supplementary table 2**: trends in in-hospital mortality for patients discharged from hospital with a main diagnosis of acute myocardial infarction and who survived over 3 days, Switzerland and Swiss regions, for period 1998-2008.

|  | **Switzerland** | **Leman** | **Mittelland** | **Northwest** | **Zurich** | **Eastern** | **Central** | **Ticino** |
| --- | --- | --- | --- | --- | --- | --- | --- | --- |
| In-hospital mortality § |  |  |  |  |  |  |  |  |
| Seven-day | 1.01 (1.00-1.03) | 1.01 (0.97-1.05) | 1.01 (0.98-1.05) | 1.02 (0.98-1.05) | 1.00 (0.96-1.03) | 1.00 (0.96-1.04) | 1.05 (0.99-1.11) | DNC |
| Overall | 1.00 (0.99-1.01) | 1.00 (0.98-1.02) | 1.00 (0.98-1.02) | 1.02 (1.00-1.05) | 0.99 (0.96-1.01) | 0.98 (0.95-1.01) | 1.04 (1.00-1.08) | 1.00 (0.96-1.05) |
| In-hospital mortality §§ |  |  |  |  |  |  |  |  |
| Seven-day | 1.02 (1.00-1.04) | 1.01 (0.97-1.05) | 1.02 (0.99-1.06) | 1.03 (1.00-1.08) | 1.00 (0.96-1.03) | 1.00 (0.96-1.04) | 1.07 (1.01-1.14) | DNC |
| Overall | 1.02 (1.00-1.03) | 1.02 (0.99-1.05) | 1.02 (0.99-1.05) | 1.04 (1.01-1.07) | 1.00 (0.97-1.02) | 0.98 (0.95-1.01) | 1.05 (1.00-1.09) | DNC |

Results are expressed as Odds ratio and (95% confidence interval) for a one-year increase. Statistical analysis by multivariate logistic regression adjusting for age (continuous), gender and transfer type; when assessing trends for Switzerland, a further adjustment was performed on region. DNC, model did not converge. §, among patients not transferred to another hospital; §§, among patients managed in a single hospital.

**Supplementary table 3**: trends in revascularisation procedures and in in-hospital mortality for patients discharged from hospital with a main diagnosis of acute myocardial infarction or unstable angina, Switzerland and Swiss regions, for period 1998-2008.

|  | **Switzerland** | **Leman** | **Mittelland** | **Northwest** | **Zurich** | **Eastern** | **Central** | **Ticino** |
| --- | --- | --- | --- | --- | --- | --- | --- | --- |
| Number of discharges | 152,662 | 31,883 | 31,371 | 25,025 | 26,091 | 15,919 | 8290 | 14,083 |
| ICU | 0.98 (0.97-0.99) | 1.01 (1.01-1.02) | 1.00 (0.99-1.01) | 0.93 (0.92-0.94) | 0.92 (0.91-0.93) | 1.09 (1.07-1.10) | 1.10 (1.08-1.12) | 0.81 (0.80-0.83) |
| Angioplasty | 1.21 (1.20-1.21) | 1.15 (1.14-1.16) | 1.18 (1.17-1.19) | 1.25 (1.24-1.27) | 1.21 (1.20-1.23) | 1.54 (1.50-1.57) | 1.76 (1.69-1.83) | 1.11 (1.10-1.13) |
| Bare stent | 1.10 (1.09-1.11) | 1.11 (1.10-1.12) | 1.06 (1.04-1.07) | 1.04 (1.02-1.05) | 1.08 (1.07-1.09) | 1.28 (1.24-1.33) | 1.60 (1.54-1.67) | 1.09 (1.07-1.11) |
| Drug-eluting stent | 1.72 (1.70-1.73) | 1.60 (1.56-1.63) | 1.60 (1.57-1.63) | 1.56 (1.53-1.59) | 2.02 (1.95-2.09) | 2.12 (2.02-2.23) | 2.97 (2.58-3.41) | 2.59 (2.45-2.73) |
| CABG | 1.00 (0.99-1.00) | 0.97 (0.95-0.98) | 1.06 (1.03-1.08) | 1.07 (1.04-1.10) | 0.97 (0.95-0.98) | DNC | 1.10 (1.02-1.19) | 0.98 (0.96-1.00) |
| Circulatory assistance | 1.16 (1.14-1.18) | 1.11 (1.07-1.16) | 1.27 (1.21-1.34) | 1.27 (1.18-1.36) | 1.11 (1.08-1.14) | 2.14 (1.63-2.81) | 1.97 (1.49-2.61) | 1.04 (0.91-1.19) |
| Thrombolysis | 1.00 (0.99-1.01) | 1.02 (1.00-1.04) | 1.00 (0.98-1.02) | 0.94 (0.91-0.97) | 1.03 (1.01-1.06) | 1.09 (1.04-1.14) | 0.98 (0.92-1.04) | 0.90 (0.86-0.94) |
| In-hospital mortality § |  |  |  |  |  |  |  |  |
| Seven-day | 1.02 (1.01-1.03) | 1.00 (0.98-1.02) | 1.03 (1.01-1.05) | 1.01 (0.99-1.03) | 1.01 (0.99-1.03) | 1.01 (0.99-1.03) | 1.03 (1.00-1.06) | DNC |
| Overall | 1.02 (1.01-1.02) | 1.00 (0.98-1.02) | 1.03 (1.01-1.05) | 1.02 (1.00-1.04) | 1.01 (0.99-1.03) | 1.00 (0.98-1.02) | 1.03 (1.00-1.06) | 1.05 (1.01-1.08) |
| In-hospital mortality §§ |  |  |  |  |  |  |  |  |
| Seven-day | 1.02 (1.01-1.03) | 1.00 (0.98-1.02) | 1.04 (1.02-1.06) | 1.01 (0.99-1.04) | 1.01 (0.99-1.03) | 1.01 (0.99-1.04) | 1.04 (1.01-1.08) | DNC |
| Overall | 1.02 (1.01-1.03) | 1.00 (0.99-1.02) | 1.04 (1.02-1.06) | 1.03 (1.01-1.05) | 1.01 (0.99-1.03) | 1.01 (0.99-1.03) | 1.04 (1.01-1.07) | 1.07 (1.03-1.10) |

Results are expressed as Odds ratio and (95% confidence interval) for a one-year increase. Statistical analysis by multivariate logistic regression adjusting for age (continuous), gender and transfer type; when assessing trends for Switzerland, a further adjustment was performed on region. ICU: intensive care unit; CABG, coronary artery bypass graft; DNC, model did not converge. §, among patients not transferred to another hospital; §§, among patients managed in a single hospital.
